# The association between frailty and in-hospital mortality in critically ill patients with congestive heart failure: results from MIMIC-IV database

**DOI:** 10.3389/fcvm.2024.1361542

**Published:** 2024-05-28

**Authors:** Dongsheng Su, Fengyun Wang, Yanhua Yang, Yinchuan Zhu, Tong Wang, Keyang Zheng, Jianmin Tang

**Affiliations:** ^1^ Department of Cardiology, Second Affiliated Hospital of Zhengzhou University, Zhengzhou, China; ^2^ Centre of Hypertension, Beijing Anzhen Hospital, Capital Medical University, Beijing, China

**Keywords:** frailty, hospital frailty risk score, congestive heart failure, in-hospital mortality, MIMIC-IV database

## Abstract

**Background:**

Frailty correlates with adverse outcomes in many cardiovascular diseases and is prevalent in individuals with heart failure (HF). The Hospital Frailty Risk Score (HFRS) offers an integrated, validated solution for frailty assessment in acute care settings, but its application in critically ill patients with congestive HF lacks exploration. This study aimed to identify the association between frailty assessed by the HFRS and in-hospital mortality in critically ill patients with congestive HF.

**Methods:**

This observational study retrospectively enrolled 12,179 critically ill patients with congestive HF. Data from the Medical Information Mart for Intensive Care IV database was used. The HFRS was calculated to assess frailty. Patients were categorized into three groups: non-frailty (HFRS < 5, *n* = 7,961), pre-frailty (5 ≤ HFRS < 15, *n* = 3,684), and frailty (HFRS ≥ 15, *n* = 534). Outcomes included in-hospital mortality, length of intensive care unit stay, and length of hospital stay. Multiple logistic regression and Locally Weighted Scatterplot Smoothing (LOWESS) smoother were used to investigate the association between frailty and outcomes. Subgroup analysis was employed to elucidate the correlation between frailty levels and in-hospital mortality across diverse subgroups.

**Results:**

12,179 patients were enrolled, 6,679 (54.8%) were male, and the average age was 71.05 ± 13.94 years. The overall in-hospital mortality was 11.7%. In-hospital mortality increased with the escalation of frailty levels (non-frailty vs. pre-frailty vs. frailty: 9.7% vs. 14.8% vs. 20.2%, *P* < 0.001). The LOWESS curve demonstrated that the HFRS was monotonically positively correlated with in-hospital mortality. Upon controlling for potential confounders, both pre-frailty and frailty statuses were found to be independently linked to a heightened risk of mortality during hospitalization (odds ratio [95% confidence interval]: pre-frailty vs. non-frailty: 1.27 [1.10–1.47], *P* = 0.001; frailty vs. non-frailty: 1.40 [1.07–1.83], *P* = 0.015; *P* for trend < 0.001). Significant interactions between frailty levels and in-hospital mortality were observed in the following subgroups: race, heart rate, creatinine, antiplatelet drug, diabetes, cerebrovascular disease, chronic renal disease, and sepsis.

**Conclusion:**

In critically ill patients with congestive HF, frailty as assessed by the HFRS emerged as an independent predictor for the risk of in-hospital mortality. Prospective, randomized studies are required to determine whether improvement of frailty levels could improve clinical prognosis.

## 1 Introduction

Frailty is defined as a condition marked by increased vulnerability to stressors, leading to a heightened risk of various adverse outcomes such as death, significant cardiovascular incidents, hospital admissions, falls, and bone fractures (1). Globally, the prevalence of frailty is estimated to be 12%, rising substantially in those with cardiovascular diseases (2). Meta-analytic studies have shown that around 17.9% of individuals with cardiovascular diseases and nearly 30% of those experiencing acute coronary syndrome are impacted by frailty (3, 4). Furthermore, a number of studies have established a connection between frailty and negative outcomes in diverse cardiocerebrovascular disorders, including acute myocardial infarction (5), acute stroke (6), and atrial fibrillation (7). The presence of frailty is notably frequent among patients with heart failure (HF) due to shared pathophysiological features, such as comorbidities, aging-related changes, and recurrent hospital admissions. These factors contribute to a decline in functional abilities and expedite the onset of sarcopenia (8). The co-occurrence of frailty and HF has been associated with diminished patient-reported and clinical outcomes (9, 10). As a result, there is a growing focus on the integration of frailty evaluations into the prognostic and therapeutic strategies for HF, aiming for a more holistic management approach (11, 12).

A significant advancement in frailty assessment emerged in the mid-1990s when it was established that combining indicators of frailty, such as slow walking speed and weight loss, into composite scores improved the prediction of adverse clinical outcomes compared to evaluating individual components separately (13, 14). Fried and colleagues further advanced this field in 2001 by introducing a pioneering frailty phenotype measurement based on five physical components, establishing a new benchmark in frailty assessment (15), which remains foundational in frailty research today (16, 17). Currently, in both epidemiological studies and clinical evaluations, more than 20 tools are utilized for frailty assessment (18). A significant development in this field is the Hospital Frailty Risk Score (HFRS), innovated and validated by Gilbert et al. This instrument employs diagnostic codes from the Tenth Revision of the International Statistical Classification of Diseases and Related Health Problems (ICD-10) (19). Its design facilitates easy integration into existing hospital electronic data systems and reduces the variability and effort usually associated with traditional frailty assessment methods. Originally designed and verified for use with patients aged 75 years and older in acute care settings, the HFRS's ability to predict outcomes has since been affirmed in various patient groups (20–23). To date, there has been limited exploration into the use of HFRS specifically for patients with congestive HF. The objective of this research was to investigate the association between frailty, assessed using the HFRS, and the incidence of mortality during hospitalization in patients with critical congestive HF.

## 2 Methods

### 2.1 Study design

**Figure 1:**
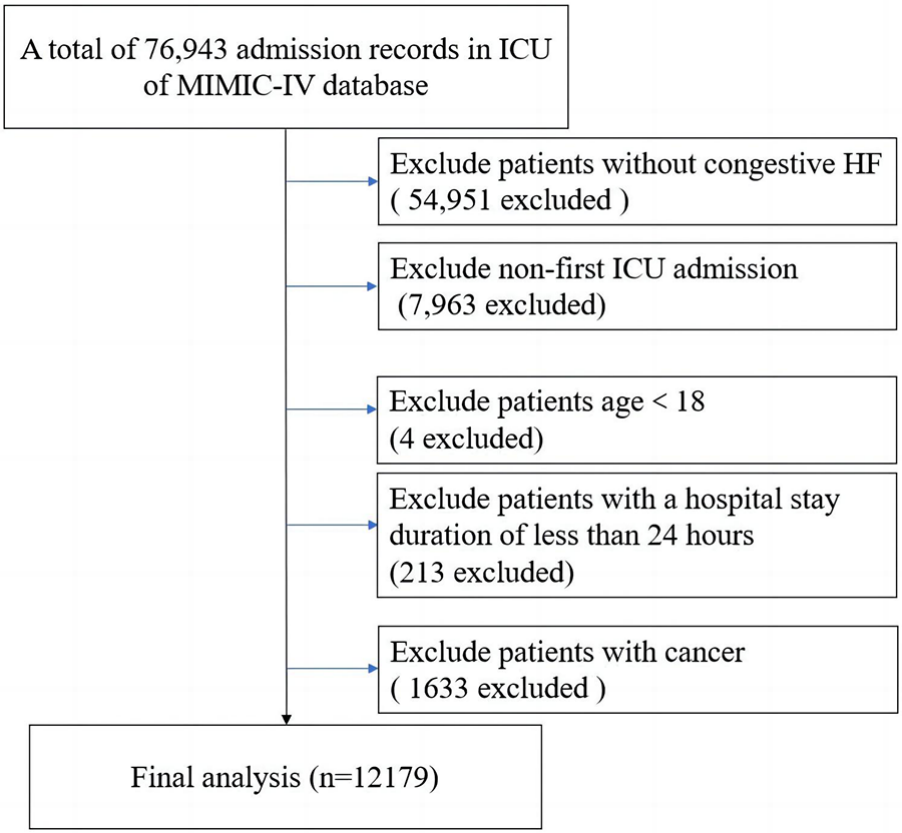
Flowchart of the study. ICU, intensive care unit; HF, heart failure.

This research constituted an observational and retrospective analysis, involving the enrollment of patients diagnosed with congestive HF. Patients meeting any of the following criteria were excluded: (1) patients without congestive HF; (2) non-first intensive care unit (ICU) admission; (3) age <18; (4) patients with a hospital stay duration of less than 24 h; (5) patients with cancer. Eventually, 12,179 patients were enrolled in our study (Figure 1).

### 2.2 Data extraction

In this research, the data were obtained from the Medical Information Mart for Intensive Care IV (MIMIC-IV) database, certified under the number 10713670. This accessible relational database, managed by the Laboratory for Computational Physiology at the Massachusetts Institute of Technology, Cambridge, MA, USA, aggregates extensive patient data in critical care settings, specifically from the ICU of Beth Israel Deaconess Medical Center, Boston, MA, USA, covering the years 2008–2019 (24, 25). For accurate disease categorization, both ICD-9 and ICD-10 diagnostic codes were employed in the MIMIC-IV database. Key variables in this study comprised age, gender, racial and ethnic backgrounds (including White, Black, Hispanic, Asian, and others), several scoring indices like the Hepatic Fibrosis and Renal Safety (HFRS), Oxford Acute Severity of Illness Score (OASIS), Simplified Acute Physiology Score II (SAPS II), Sequential Organ Failure Assessment (SOFA), and Systemic Inflammatory Response Syndrome (SIRS) scores, along with laboratory data, treatment modalities, and patient comorbidities.

### 2.3 Assessment of frailty, grouping and outcomes

In this study, the HFRS was employed to evaluate frailty levels in patients with critical illnesses and congestive HF. The HFRS, a novel and validated tool for frailty assessment, utilizes ICD-10 diagnostic codes to identify patients potentially facing adverse healthcare outcomes. Following the methodology described by Gilbert et al., weights were assigned to 109 specific ICD-10 codes, with the total HFRS being the aggregate of these codes (19). For individual patients, the HFRS was computed using one or more of the 109 ICD-10-CM diagnosis codes noted at the time of admission for their initial hospital stay (5). Patients were classified into three frailty categories based on the HFRS scores: non-frailty (HFRS less than 5), pre-frailty (HFRS between 5 and 14), and frailty (HFRS 15 or higher). We analyzed mortality during hospitalization, length of stay in the ICU, and the overall duration of hospitalization as key outcomes to explore the correlation between frailty (as determined by the HFRS) and these clinical outcomes.

### 2.4 Statistical analysis

Baseline patient data were presented by calculating means and standard deviations for data following a normal distribution, median and interquartile ranges for data that were not normally distributed, and numbers and percentages for categorical data. Patient features across different levels of frailty were compared employing various statistical methods, including analysis of variance, the Kruskal–Wallis test, and the Chi-square test, depending on their suitability. To investigate the link between varying degrees of frailty and mortality during hospitalization, multiple logistic regression analysis was utilized in models 1, 2, and 3. The findings were reported as odds ratios (OR) with 95% confidence intervals (CI). In model 3, confounders were selected using a stepwise approach, with variables being excluded if their *P*-value exceeded 0.05. Besides, Locally Weighted Scatterplot Smoothing (LOWESS) smoother was applied to examine the relationship between the HFRS on a continuous scale and the risk of mortality during hospitalization (the bandwidth was set to 0.8). Subgroup analyses were performed to evaluate the impact of the HFRS on mortality during hospital stays across various subgroups. These included factors such as age, gender, race, heart rate, body mass index, systolic and diastolic blood pressure, hemoglobin, creatinine, vasopressor drugs, antiplatelet drugs, diabetes, cerebrovascular disease, acute kidney injury, chronic renal disease, and sepsis. *P* for interaction was calculated. Among the continuous variables, age was grouped using 65 years as the threshold, while the remaining continuous variables were divided into subgroups according to their respective median values. To manage missing data in our study, we employed multiple imputation techniques. All tests were two-sided. Statistical significance was set at *P* < 0.05. All statistical analyses were performed utilizing the R software (R-project®; R Foundation for Statistical Computing, Vienna, Austria, Ver. 4.2.1).

## 3 Results

### 3.1 Subjects and baseline characteristics

In total, 12,179 patients were included in the study, with a mean age of 71.05 ± 13.94 years, among which 6,679 (54.8%) were males. Participants were segmented into three categories based on their frailty status: non-frailty (*n* = 7,961), pre-frailty (*n* = 3,684), and frailty (*n* = 534). Table 1 displays the baseline features of each group. Patients with higher levels of frailty tended to be older, with fewer females, lower body mass index, faster heart rate, and higher scores across all scales.

**Table 1:** Characteristics of participants with congestive heart failure in MIMIC-IV database stratified according to frailty levels.

Characteristics	Overall (*n* = 12,179)	Non-frailty (*n* = 7,961)	Pre-frailty (*n* = 3,684)	Frailty (*n* = 534)	*P* Value
Age, y	71.05 ± 13.94	69.74 ± 14.06	72.99 ± 13.59	77.23 ± 11.02	<0.001
Sex, *n* (%)					<0.001
Male	6,679 (54.8)	4,545 (57.1)	1,873 (50.8)	261 (48.9)	
Female	5,500 (45.2)	3,416 (42.9)	1,811 (49.2)	273 (51.1)	
Race, *n* (%)					0.001
White	8,436 (69.3)	5,412 (68.0)	2,640 (71.7)	384 (71.9)	
Black	1,264 (10.4)	825 (10.4)	382 (10.4)	57 (10.7)	
Hispanic	348 (2.9)	247 (3.1)	91 (2.5)	10 (1.9)	
Asian	298 (2.4)	204 (2.6)	81 (2.2)	13 (2.4)	
Other	1,833 (15.1)	1,273 (16.0)	490 (13.3)	70 (13.1)	
Body mass index, kg/m^2^	29.10 ± 8.56	29.30 ± 8.45	28.86 ± 8.69	27.89 ± 9.17	<0.001
Vital signs					
Heart rate, bpm	87.00 ± 19.84	86.61 ± 19.75	87.41 ± 19.78	90.05 ± 21.22	<0.001
Systolic blood pressure, mm Hg	122.56 ± 24.64	122.35 ± 24.37	122.68 ± 24.98	124.77 ± 26.21	0.084
Diastolic blood pressure, mm Hg	66.33 ± 18.22	67.26 ± 17.88	64.37 ± 18.58	66.01 ± 19.61	<0.001
Scales, points					
HFRS	2.5 [0, 7.1]	0 [0, 2.2]	8.5 [6.6, 10.9]	17.4 [16.1, 20.0]	<0.001
OASIS	32 [26, 38]	31 [25, 37]	34 [27, 40]	36 [31, 42]	<0.001
SAPS II	37 [30, 45]	35 [29, 44]	39 [32, 48]	43 [36, 51]	<0.001
SOFA	5 [3, 8]	4 [2, 7]	5 [3, 8]	6 [4, 8]	<0.001
Laboratory parameters					
Lymphocyte percent, %	12.14 ± 7.30	12.51 ± 7.48	11.48 ± 6.82	11.26 ± 7.45	<0.001
Neutrophil percent, %	79.00 ± 9.11	78.40 ± 8.99	80.12 ± 9.20	80.26 ± 9.46	<0.001
Platelet, 10^9^/L	213.10 ± 97.86	208.75 ± 91.14	221.08 ± 107.99	222.86 ± 115.55	<0.001
White blood cell, 10^9^/L	10.84 ± 5.75	10.68 ± 5.52	11.11 ± 6.13	11.24 ± 6.40	<0.001
Red blood cell, 10^9^/L	3.66 ± 0.76	3.69 ± 0.77	3.61 ± 0.72	3.56 ± 0.72	<0.001
Hemoglobin, g/dl	12.14 ± 7.30	12.51 ± 7.48	11.48 ± 6.82	11.26 ± 7.45	<0.001
pH	7.38 ± 0.08	7.38 ± 0.08	7.37 ± 0.09	7.37 ± 0.10	<0.001
PaCO2, mm Hg	43.44 ± 11.47	43.26 ± 10.75	44.00 ± 12.97	42.24 ± 10.78	<0.001
PaO2, mm Hg	100 [70, 176]	100 [69, 203]	100 [74, 151]	100 [66, 123]	<0.001
LDH, mg/L	1.6 [1.3, 2.0]	1.6 [1.3, 1.9]	1.6 [1.2, 2.0]	1.6 [1.2, 2.2]	0.163
SpO2, %	87.15 ± 12.31	86.65 ± 12.67	88.16 ± 11.46	87.75 ± 11.97	<0.001
Albumin, g/dl	3.28 ± 0.49	3.32 ± 0.48	3.22 ± 0.50	3.13 ± 0.49	<0.001
Blood urea Nitrogen, mg/dl	26 [17, 41]	23 [17, 37]	31 [20, 48]	31 [21, 50]	<0.001
Creatinine, mg/dl	1.2 [0.9, 1.8]	1.1 [0.8, 1.6]	1.4 [0.9, 2.1]	1.4 [0.9, 2.1]	<0.001
Glucose, mg/dl	124 [102, 162]	123 [102, 159]	124 [101, 166]	128 [105, 173]	0.126
Potassium, mmol/L	4.27 ± 0.73	4.28 ± 0.71	4.26 ± 0.77	4.18 ± 0.77	0.004
Sodium, mmol/L	138.53 ± 5.03	138.56 ± 4.76	138.33 ± 5.40	139.43 ± 6.20	<0.001
Treatment, *n* (%)					
Vasopressin	939 (7.7)	603 (7.6)	291 (7.9)	45 (8.4)	0.678
Dopamine	671 (5.5)	322 (4.0)	303 (8.2)	46 (8.6)	<0.001
Epinephrine	1,153 (9.5)	882 (11.1)	258 (7.0)	13 (2.4)	<0.001
Norepinephrine	2,899 (23.8)	1,828 (23.0)	924 (25.1)	147 (27.5)	0.005
Aspirin	8,769 (72.0)	5,849 (73.5)	2,560 (69.5)	360 (67.4)	<0.001
Clopidogrel	2,508 (20.6)	1,695 (21.3)	726 (19.7)	87 (16.3)	0.006
Statins	8,207 (67.4)	5,564 (69.9)	2,311 (62.7)	332 (62.2)	<0.001
Ticagrelor	205 (1.7)	179 (2.2)	26 (0.7)	0 (0.0)	<0.001
Prasugrel	72 (0.6)	58 (0.7)	14 (0.4)	0 (0.0)	0.014
Beta-blockers	10,150 (83.3)	6,635 (83.3)	3,068 (83.3)	447 (83.7)	0.969
ACEI/ARB	5,546 (45.5)	3,768 (47.3)	1,568 (42.6)	210 (39.3)	<0.001
Thiazide diuretics	1,167 (9.6)	768 (9.6)	349 (9.5)	50 (9.4)	0.942
Loop diuretics	9,964 (81.8)	6,514 (81.8)	3,014 (81.8)	436 (81.6)	0.995
MRA	1,146 (9.4)	749 (9.4)	356 (9.7)	41 (7.7)	0.34
Digitalis	1,333 (10.9)	806 (10.1)	455 (12.4)	72 (13.5)	<0.001
Corticosteroids	4,173 (34.3)	2,637 (33.1)	1,365 (37.1)	171 (32.0)	<0.001
Antibiotics	9,751 (80.1)	6,082 (76.4)	3,162 (85.8)	507 (94.9)	<0.001
Oral anticoagulants	4,894 (40.2)	3,332 (41.9)	1,380 (37.5)	182 (34.1)	<0.001
Dialysis	1,108 (9.1)	640 (8.0)	414 (11.2)	54 (10.1)	<0.001
ECMO	65 (0.5)	41 (0.5)	24 (0.7)	0 (0.0)	0.144
Mechanical ventilation	4,479 (36.8)	2,830 (35.5)	1,438 (39.0)	211 (39.5)	0.001
Comorbidities, *n* (%)					
Essential hypertension	3,707 (30.4)	2,145 (26.9)	1,377 (37.4)	185 (34.6)	<0.001
Diabetes	5,051 (41.5)	3,180 (39.9)	1,629 (44.2)	242 (45.3)	<0.001
Cerebrovascular disease	1,747 (14.3)	1,005 (12.6)	562 (15.3)	180 (33.7)	<0.001
Myocardial infarction	4,005 (32.9)	2,829 (35.5)	1,041 (28.3)	135 (25.3)	<0.001
Pulmonary embolism	599 (4.9)	413 (5.2)	146 (4.0)	40 (7.5)	<0.001
Dyslipidemia	6,394 (52.5)	4,379 (55.0)	1,759 (47.7)	256 (47.9)	<0.001
Atrial fibrillation	6,859 (56.3)	4,341 (54.5)	2,184 (59.3)	334 (62.5)	<0.001
Acute kidney injury	8,713 (71.5)	5,436 (68.3)	2,841 (77.1)	436 (81.6)	<0.001
Chronic renal disease	4,603 (37.8)	2,670 (33.5)	1,657 (45.0)	276 (51.7)	<0.001
Dementia	606 (5.0)	408 (5.1)	85 (2.3)	113 (21.2)	<0.001
Sepsis	6,515 (53.5)	3,748 (47.1)	2,368 (64.3)	399 (74.7)	<0.001

ACEI, angiotensin-converting enzyme inhibitor; ARB, angiotensin receptor blocker; ECMO, extracorporeal membrane oxygenation; HFRS, hospital frailty risk score; LDH, lactate dehydrogenase; MRA, aldosterone receptor antagonist; OASIS, Oxford acute severity of illness score; PaCO2, partial pressure of carbon dioxide in artery; PaO2, partial pressure of oxygen in artery; pH, potential of hydrogen; SAPS II, simplified acute physiology score II; SOFA, sequential organ failure assessment; SpO2, saturation of peripheral oxygen.

Neutrophil, platelet, white blood cell, blood urea nitrogen, and creatinine levels rose with the escalation of frailty level, while lymphocyte, red blood cell, hemoglobin, pH, albumin, and potassium levels declined as the level of frailty rose. Additionally, patients with higher levels of frailty received more treatment, such as dopamine, epinephrine, norepinephrine, aspirin, clopidogrel, statins, ticagrelor, prasugrel, angiotensin-converting enzyme inhibitor/angiotensin receptor blocker, digitalis, corticosteroids, antibiotics, oral anticoagulants, warfarin, factor Xa inhibitors, heparin, low molecular weight heparin, dialysis, and mechanical ventilation. A greater proportion of frail patients had complications, such as essential hypertension, diabetes, cerebrovascular disease, myocardial infarction, cardiomyopathy, valve disease, pulmonary embolism, dyslipidemia, atrial fibrillation, cardiogenic shock, septic shock, acute kidney injury, chronic renal disease, dementia, and sepsis.

### 3.2 Associations between frailty levels and outcomes

Clinical outcome events are illustrated in Table 2. The overall in-hospital mortality was 11.7%. In-hospital mortality significantly increased with the escalation of frailty levels [OR (95% CI): non-frailty vs. pre-frailty vs. frailty: 9.7% vs. 14.8% vs. 20.2%, *P* < 0.001]. In addition, the length of ICU stay (OR [95% CI]: non-frailty vs. pre-frailty vs. frailty: 2.17 [1.23–4.03] vs. 2.76 [1.44–5.34] vs. 3.03 [1.65–6.63], *P* < 0.001) and the length of hospital stay (OR [95% CI]: non-frailty vs. pre-frailty vs. frailty: 7.63 [4.79–12.63] vs. 8.75 [5.59–13.96] vs. 11.42 [6.83–17.98], *P* < 0.001) increased as the level of frailty rose.

**Table 2:** Association between frailty levels and all outcomes.

Outcomes	Overall (*n* = 12,179)	Non-frailty (*n* = 7,961)	Pre-frailty (*n* = 3,684)	Frailty (*n* = 534)	*P* Value
In-hospital mortality, *n* (%)	1,423 (11.7)	771 (9.7)	544 (14.8)	108 (20.2)	<0.001
Length of ICU stay, days	2.30 [1.28, 4.50]	2.17 [1.23, 4.03]	2.76 [1.44, 5.34]	3.03 [1.65, 6.63]	<0.001
Length of hospital stay, days	8.00 [5.01, 13.12]	7.63 [4.79, 12.63]	8.75 [5.59, 13.96]	11.42 [6.83, 17.98]	<0.001

ICU, intensive care unit.

The impact of frailty on endpoints was explored through multiple logistic regression analyses, as detailed in Table 3. As indicated by model 1, both pre-frailty and frailty statuses showed a correlation with an increased risk of mortality during hospitalization (OR [95% CI]: pre-frailty vs. non-frailty: 1.62 [1.44–1.82], *P* < 0.001; frailty vs. non-frailty: 2.36 [1.89–2.96], *P* < 0.001; *P* for trend <0.001). Upon analyzing the HFRS as a continuous variable, each incremental unit increase was associated with a heightened risk of mortality during hospitalization (OR [95% CI]: 1.04 [1.03–1.06], *P* < 0.001). In model 2, factors such as age, gender, and race were included. The greatest level of frailty was consistently associated with the most elevated risk of mortality during hospital stays (OR [95% CI]: pre-frailty vs. non-frailty 1.55 [1.38–1.75], *P* < 0.001; frailty vs. non-frailty: 2.08 [1.66–2.61], *P* < 0.001; *P* for trend <0.001). In model 2, the HFRS was consistent with model 1 when analyzed as a continuous variable (OR [95% CI]: 1.04 [1.03–1.05], *P* < 0.001). Model 3 added more variables which were obtained through stepwise regression. Both pre-frailty and frailty statuses continued to demonstrate an association with the risk of mortality during hospitalization (OR [95% CI]: pre-frailty vs. non-frailty: 1.27 [1.10–1.47], *P* = 0.001; frailty vs. non-frailty: 1.40 [1.07–1.83], *P* = 0.015; *P* for trend <0.001). In model 3, when evaluating the HFRS as a continuous measure, each incremental increase in the score was independently linked to a higher risk of mortality during hospitalization (OR [95% CI]: 1.01 [1.00–1.03], *P* = 0.018).

**Table 3:** The association between frailty and in-hospital mortality.

	OR (95% CI)	*P* Value	*P* for trend
Model 1			<0.001
Non-frailty	Reference		
Pre-frailty	1.62 (1.44–1.82)	<0.001	
Frailty	2.36 (1.89–2.96)	<0.001	
Continuous	1.04 (1.03–1.06)	<0.001	
Model 2			<0.001
Non-frailty	Reference		
Pre-frailty	1.55 (1.38–1.75)	<0.001	
Frailty	2.08 (1.66–2.61)	<0.001	
Continuous	1.04 (1.03–1.05)	<0.001	
Model 3			<0.001
Non-frailty	Reference		
Pre-frailty	1.27 (1.10–1.47)	0.001	
Frailty	1.40 (1.07–1.83)	0.015	
Continuous	1.01 (1.00–1.03)	0.018	

ACEI, angiotensin-converting enzyme inhibitor; ARB, angiotensin receptor blocker; CI, confidence interval; ECMO, extracorporeal membrane oxygenation; LDH, lactate dehydrogenase; OR, odds ratio; PaO2, partial pressure of oxygen in artery.

Models were derived from binary logistic regression analysis.

Model 1: unadjusted.

Model 2: adjusted for age, gender, ethnicity.

Model 3: adjusted for age, gender, ethnicity, heart rate, lymphocyte percent, platelet, PaO2, LDH, albumin, creatinine, vasopressin, dopamine, norepinephrine, phenylephrine, milrinone, sspirin, statins, beta-blockers, ACEI/ARB, thiazide diuretics, corticosteroids, oral anticoagulants, factor Xa inhibitors, heparin, dialysis, ECMO, cerebrovascular disease, myocardial infarction, dyslipidemia, pulmonary embolism, atrial fibrillation, ventricular arrhythmias, ventricular fibrilation, cardiogenic shock, septic shock.

**Figure 2:**
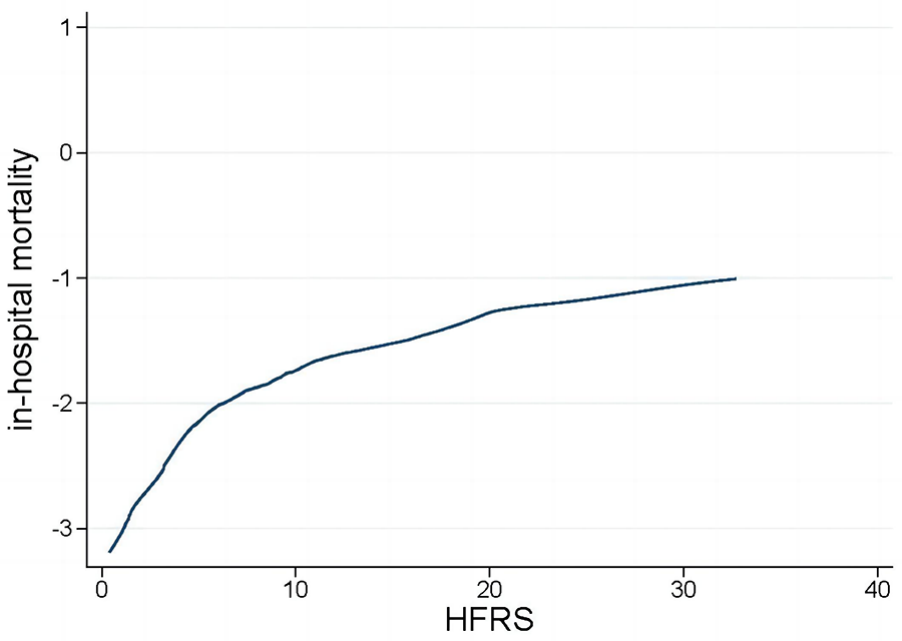
Locally weighted scatterplot smoothing curve. HFRS, hospital frailty risk score.

The LOWESS curve depicted in Figure 2 illustrates a consistent positive correlation between the HFRS and the risk of in-hospital mortality among patients who are critically ill with congestive HF.

### 3.3 Subgroup analysis

**Figure 3:**
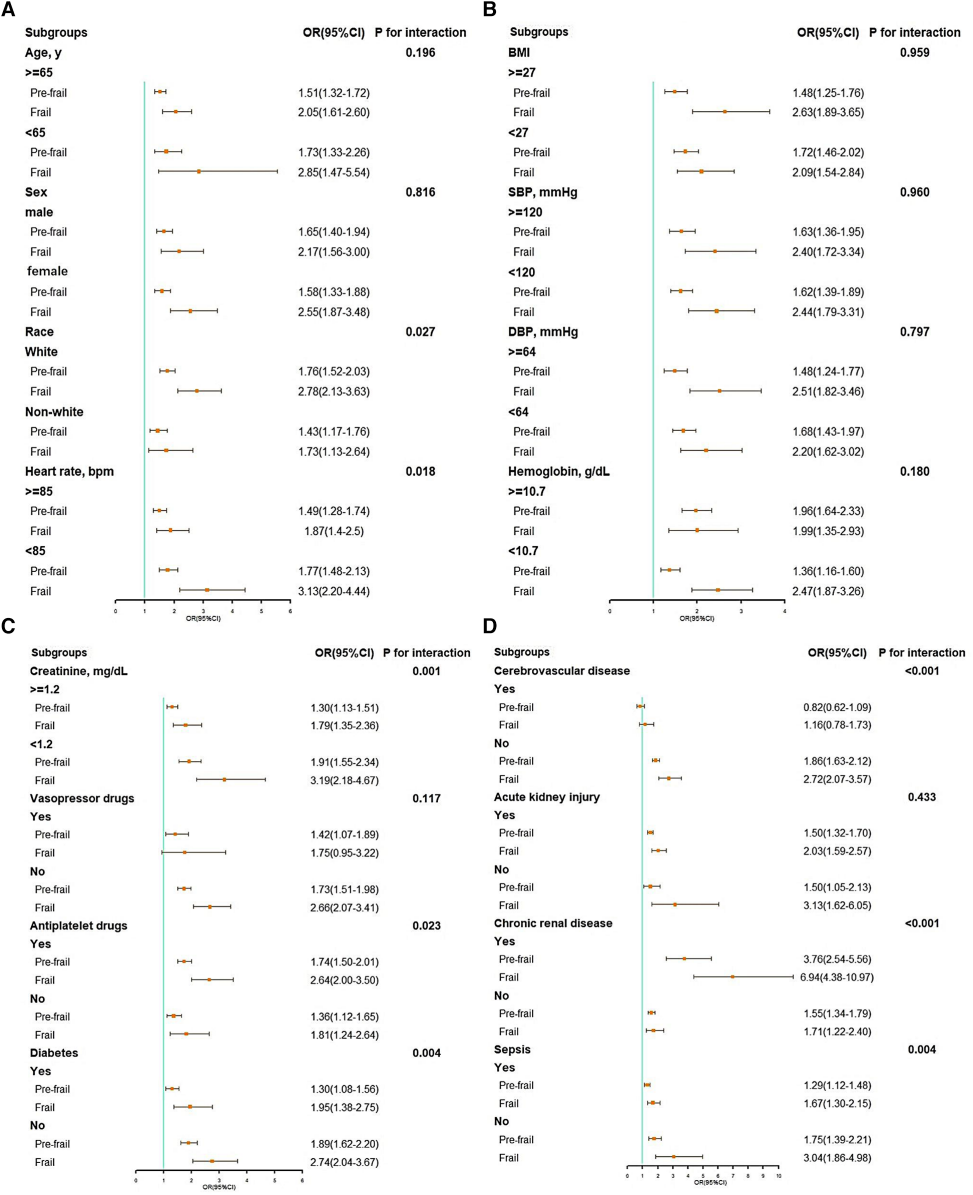
Subgroup analysis of associations between in-hospital mortality and the hospital frailty risk score. (**A**) Subgroup analyses: age, gender, race, and heart rate; (**B**) subgroup analyses: BMI, SBP, DBP, and hemoglobin; (**C**) subgroup analyses: creatinine, vasopressor drugs, antiplatelet drugs, and diabetes; (**D**) subgroup analyses: cerebrovascular disease, acute kidney injury, chronic renal disease, and sepsis. BMI, body mass index; CI, confidence interval; DBP, diastolic blood pressure; OR, odds ratio; SBP, systolic blood pressure.

A significant interaction was observed in subgroups of race (*P* = 0.027 for interaction), heart rate (*P* = 0.018 for interaction), creatinine (*P* < 0.001 for interaction), antiplatelet drug (*P* = 0.023 for interaction), diabetes (*P* = 0.004 for interaction), cerebrovascular disease (*P* < 0.001 for interaction), chronic renal disease (*P* < 0.001 for interaction), and sepsis (*P* = 0.004 for interaction) (see Figure 3).

## 4 Discussion

In this study, we investigated the relationship between frailty as assessed by the HFRS and the prognosis in critically ill patients with congestive HF. We found frailty levels were associated with higher in-hospital mortality, length of ICU stay, and length of hospital stay. Moreover, pre-frailty and frailty emerged as independent risk factors for in-hospital mortality in patients with congestive HF, even after adjusting for possible confounding variables. Taken together, frailty accessed by the HFRS can be used to identify patients with congestive HF who are at higher risk of disability and adverse clinical outcomes, and hence facilitate targeted interventions that reduce frailty burden and improve outcomes.

Frailty is prevalent in individuals with HF, with a prevalence of 56% to 76% among hospitalized patients with HF (8). Frail individuals with HF have a greater symptom burden, including dyspnea, sleep disturbances, and depressive symptoms, when compared to their non-frail counterparts (26). The quality of life is also markedly inferior in frail vs. non-frail patients with chronic and acute HF (27). A meta-analysis revealed that frail individuals with HF had a 57% elevated risk of hospitalization and an 80% increased risk of mortality in comparison to their non-frail counterparts (28). Another study showed that frailty was significantly associated with a higher risk of HF hospitalization and all-cause mortality in patients with HF (29).

With over 20 frailty assessment tools available (18), selecting the most appropriate one for varied clinical environments presents a significant challenge for healthcare professionals. Additionally, the optimal timing to assess frailty levels in an acute scenario is a subject of debate. Furthermore, it is important to note that current methods for assessing frailty largely rely on manual scoring systems (18), which can place a considerable workload on medical practitioners. Adopting the HFRS in clinical settings could proficiently overcome the previously mentioned difficulties associated with routine frailty evaluations in medical practice. The HFRS seamlessly integrates into electronic medical records, allowing for immediate calculation following the completion of the diagnosis process. The HFRS has been rigorously validated both internally and externally, utilizing administrative data across a variety of patient demographics and international settings. To the best of our knowledge, this research is the first attempt to assess the predictive significance of the HFRS in patients with critical congestive HF. Our results demonstrated that the HFRS is an effective prognostic tool for predicting in-hospital mortality among critically ill patients with congestive HF and could be used in clinical practice. This enhanced the overall risk evaluation and stratification process, complementing traditional risk factors for a more thorough assessment. Additionally, the LOWESS curve indicated a positive, linear correlation between the HFRS and the risk of in-hospital mortality: a notable increase in mortality risk was observed with rising HFRS levels. This suggested that medical professionals could potentially mitigate frailty through intensified treatment and enhanced care, thereby improving clinical outcomes and lessening both physical and financial impacts on patients.

In addition, the findings from our subgroup analyses provided valuable insights. The association between the HFRS and mortality during hospitalization differed across patients with varying baseline profiles. Higher baseline heart rate, serum creatinine concentration, prevalence of diabetes, cerebrovascular disease, and sepsis are associated with elevated frailty severity. Also, we observed that the association between the HFRS and mortality during hospitalization was less pronounced in subgroups with elevated heart rate, creatinine, and complications such as diabetes, cerebrovascular disease, and sepsis. One possible reason for this could be that patients who were more frail at baseline, characterized by a higher incidence of complications and abnormalities in clinical indicators like serum results, tend to receive additional physiologic support and intensified medical care (30), potentially weakening the impact of frailty on adverse outcomes.

However, the effect of frailty on in-hospital mortality was significantly amplified in the subgroup with chronic renal disease, suggesting a synergistic effect of chronic renal disease and frailty in increasing the risk of short-term mortality. This observation underlined the urgent need for effective therapeutic interventions in this population. A recent meta-analysis of the DAPA-HF and EMPEROR-Reduced trials has elucidated the role of SGLT2 inhibition in managing HF with reduced ejection fraction, irrespective of diabetes status (31). This comprehensive analysis not only confirmed a reduction in all-cause and cardiovascular death but also highlighted improvements in renal outcomes among patients with HF and reduced ejection fraction who received SGLT2 inhibitors. Such findings are particularly relevant for the population in this study, suggesting a broader application of these treatments in frail individuals, including those with kidney disease. Furthermore, the beneficial effects of the SGLT2 inhibitor empagliflozin on cognitive and physical impairment in frail older adults with diabetes and hypertension have been demonstrated (32). These results, emphasizing the potential of SGLT2 inhibition to mitigate mitochondrial oxidative stress (32), validate the efficacy and safety of SGLT2 inhibitors in frail older adults, revealing substantial treatment benefits for this often overlooked and high-risk group (33–35). The congruence of these findings with our observations underscores the necessity of further research into the use of SGLT2 inhibitors, like empagliflozin and dapagliflozin, in patients with congestive HF and frailty, especially those with kidney disease. Further studies are needed to explore these therapeutic avenues in more detail in the future.

Frailty has long been considered a manifestation of expedited aging, involving the accumulation of age-related deficits in various physiological systems, leading to an increased susceptibility to negative outcomes (18). Likewise, HF is intimately associated with aging, exhibiting a significantly greater occurrence and prevalence among older populations. Older patients with HF often face worse clinical outcomes, more HF-related hospitalizations, and higher healthcare costs (36). Therefore, the HFRS was originally formulated and authenticated for use in people aged 75 years and older, with its external validation predominantly targeting the older demographic. However, interestingly, our research uncovered a link between the HFRS and the risk of mortality in patients with congestive HF under the age of 65 years. In fact, frailty was demonstrated to be a condition that can surface in adults at any stage of life (37), particularly among those with chronic conditions, underscoring the need for clinicians to diligently consider frailty levels in younger patients.

Our study highlights the advantages of using the HFRS in clinical settings, especially in emergency situations where a quick and cost-efficient evaluation of a patient's health is crucial. The strengths of our study include: (1) cost-efficiency and accessibility: the HFRS is known for its cost-effectiveness and widespread availability, making it an invaluable tool for clinicians under time constraints and resource limitations; (2) rapid risk identification: in emergency scenarios, the HFRS enables the swift identification of high-risk patients due to frailty, facilitating timely and potentially life-saving interventions; (3) prognostic value: our findings suggest that for patients with congestive HF and higher HFRS scores, earlier interventions to mitigate frailty could lead to improved outcomes, underscoring the prognostic value of the HFRS in clinical practice. These aspects collectively underscore the utility and importance of the HFRS in enhancing patient care, particularly in acute care settings.

While our study offers valuable insights, it is subject to certain limitations that should be considered: (1) As a retrospective analysis, our study may be influenced by inherent biases that could affect the validity and generalizability of our findings. The retrospective design limits our ability to establish causality, highlighting the need for future prospective research to validate our results in broader populations; (2) the MIMIC-IV database included missing or incomplete historical medical records, which may have led to underestimation of the HFRS for some patients. This limitation points to the need for more comprehensive data collection methods in the critical care setting; (3) the absence of critical data within the MIMIC-IV database, such as detailed causes of death, left ventricular ejection fraction measurements, and specific clinical symptoms, prevented a more thorough analysis; (4) the significant discrepancy in the number of participants assigned to each group is also one of the limitations of this study. The unequal distribution of participants may have influenced the statistical power of our analyses and could potentially affect the generalizability of the results. The inclusion of these variables in future studies could significantly enhance the understanding and management of congestive HF and frailty. By addressing these limitations in future research, we can further elucidate the role of the HFRS in managing patients with congestive HF and frailty, ultimately contributing to better patient outcomes.

## 5 Conclusion

In individuals with congestive HF, frailty, as evaluated using the HFRS, was identified as an independent predictor of the risk linked to in-hospital mortality. Future prospective, randomized studies are essential to ascertain whether improvement of frailty levels can lead to better clinical outcomes.

## Data Availability

The data used in this study were from the Medical Information Mart for Intensive Care IV (MIMIC-IV, version 2.2) database (https://physionet.org/content/mimiciv/2.2/). The authors were approved to access to the database (ID: 12353225).

## References

[1] Clegg A Young J Iliffe S Rikkert MO Rockwood K. Frailty in elderly people. Lancet. (2013) 381(9868):752–62. 10.1016/S0140-6736(12)62167-9 23395245 PMC4098658

[2] O'Caoimh R Sezgin D O'Donovan MR Molloy DW Clegg A Rockwood K Prevalence of frailty in 62 countries across the world: a systematic review and meta-analysis of population-level studies. Age Ageing. (2021) 50(1):96–104. 10.1093/ageing/afaa219 33068107

[3] Veronese N Cereda E Stubbs B Solmi M Luchini C Manzato E Risk of cardiovascular disease morbidity and mortality in frail and pre-frail older adults: results from a meta-analysis and exploratory meta-regression analysis. Ageing Res Rev. (2017) 35:63–73. 10.1016/j.arr.2017.01.003 28143778 PMC6047747

[4] Xu W Cai Y Liu H Fan L Wu C. Frailty as a predictor of all-cause mortality and readmission in older patients with acute coronary syndrome: a systematic review and meta-analysis. Wien Klin Wochenschr. (2020) 132(11–12):301–9. 10.1007/s00508-020-01650-9 32342196

[5] Bai W Hao B Meng W Qin J Xu W Qin L. Association between frailty and short- and long-term mortality in patients with critical acute myocardial infarction: results from MIMIC-IV. Front Cardiovasc Med. (2022) 9:1056037. 10.3389/fcvm.2022.1056037 36588580 PMC9797732

[6] Burton JK Stewart J Blair M Oxley S Wass A Taylor-Rowan M Prevalence and implications of frailty in acute stroke: systematic review & meta-analysis. Age Ageing. (2022) 51(3):afac064. 10.1093/ageing/afac064 35352795 PMC9037368

[7] Wilkinson C Wu J Searle SD Todd O Hall M Kunadian V Clinical outcomes in patients with atrial fibrillation and frailty: insights from the ENGAGE AF-TIMI 48 trial. BMC Med. (2020) 18(1):401. 10.1186/s12916-020-01870-w 33357217 PMC7758931

[8] Pandey A Kitzman D Reeves G. Frailty is intertwined with heart failure: mechanisms, prevalence, prognosis, assessment, and management. JACC Heart Fail. (2019) 7(12):1001–11. 10.1016/j.jchf.2019.10.005 31779921 PMC7098068

[9] Joseph SM Rich MW. Targeting frailty in heart failure. Curr Treat Options Cardiovasc Med. (2017) 19(4):31. 10.1007/s11936-017-0527-5 28357683

[10] Vitale C Spoletini I Rosano GM. Frailty in heart failure: implications for management. Card Fail Rev. (2018) 4(2):104–6. 10.15420/cfr.2018.22.2 30206485 PMC6125710

[11] Forman DE Santanasto AJ Boudreau R Harris T Kanaya AM Satterfield S Impact of incident heart failure on body composition over time in the health, aging, and body composition study population. Circ Heart Fail. (2017) 10(9):e003915. 10.1161/CIRCHEARTFAILURE.117.003915 28899988 PMC5658007

[12] Rich MW Chyun DA Skolnick AH Alexander KP Forman DE Kitzman DW Knowledge gaps in cardiovascular care of the older adult population: a scientific statement from the American heart association, American college of cardiology, and American geriatrics society. J Am Coll Cardiol. (2016) 67(20):2419–40. 10.1016/j.jacc.2016.03.004 27079335 PMC7733163

[13] Sager MA Rudberg MA Jalaluddin M Franke T Inouye SK Landefeld CS Hospital admission risk profile (HARP): identifying older patients at risk for functional decline following acute medical illness and hospitalization. J Am Geriatr Soc. (1996) 44(3):251–7. 10.1111/j.1532-5415.1996.tb00910.x 8600192

[14] Corti MC Guralnik JM Salive ME Sorkin JD. Serum albumin level and physical disability as predictors of mortality in older persons. JAMA. (1994) 272(13):1036–42. 10.1001/jama.1994.03520130074036 8089886

[15] Fried LP Tangen CM Walston J Newman AB Hirsch C Gottdiener J Frailty in older adults: evidence for a phenotype. J Gerontol A Biol Sci Med Sci. (2001) 56(3):M146–56. 10.1093/gerona/56.3.M146 11253156

[16] Rizzo M Pansini A Colucci M Boccalone E Mone P. Frailty in nursing home residents. Eur J Intern Med. (2023) 115:152–3. 10.1016/j.ejim.2023.05.027 37258383

[17] Mone P De Gennaro S Frullone S Marro A Santulli G. Hyperglycemia drives the transition from pre-frailty to frailty: the monteforte study. Eur J Intern Med. (2023) 111:135–7. 10.1016/j.ejim.2023.01.006 36635128 PMC10122706

[18] Afilalo J Alexander KP Mack MJ Maurer MS Green P Allen LA Frailty assessment in the cardiovascular care of older adults. J Am Coll Cardiol. (2014) 63(8):747–62. 10.1016/j.jacc.2013.09.070 24291279 PMC4571179

[19] Gilbert T Neuburger J Kraindler J Keeble E Smith P Ariti C Development and validation of a hospital frailty risk score focusing on older people in acute care settings using electronic hospital records: an observational study. Lancet. (2018) 391(10132):1775–82. 10.1016/S0140-6736(18)30668-8 29706364 PMC5946808

[20] Ramai D Dang-Ho KP Kewalramani A Bandaru P Sacco R Giacomelli L Hospital frailty risk score is independently associated with mortality and encephalopathy in hospitalized patients with hepatocellular carcinoma. Biomedicines. (2021) 9(11):1693. 10.3390/biomedicines9111693 34829921 PMC8615905

[21] Nghiem S Afoakwah C Scuffham P Byrnes J. Hospital frailty risk score and adverse health outcomes: evidence from longitudinal record linkage cardiac data. Age Ageing. (2021) 50(5):1778–84. 10.1093/ageing/afab073 33989395

[22] Meyer M Schwarz T Renkawitz T Maderbacher G Grifka J Weber M. Hospital frailty risk score predicts adverse events in revision total hip and knee arthroplasty. Int Orthop. (2021) 45(11):2765–72. 10.1007/s00264-021-05038-w 33860337 PMC8560670

[23] Aitken SJ Lujic S Randall DA Noguchi N Naganathan V Blyth FM. Predicting outcomes in older patients undergoing vascular surgery using the hospital frailty risk score. Br J Surg. (2021) 108(6):659–66. 10.1002/bjs.12043 34157089

[24] Chen H Gong SR Yu RG. Association between normalized lactate load and mortality in patients with septic shock: an analysis of the MIMIC-III database. BMC Anesthesiol. (2021) 21(1):16. 10.1186/s12871-021-01239-3 33435876 PMC7802303

[25] Johnson A Bulgarelli L Pollard T Horng S Celi LA Mark R. MIMIC-IV (version 2.2). PhysioNet. (2023). Available online at: 10.13026/6mm1-ek67 (Accessed September 19, 2023).

[26] Denfeld QE Winters-Stone K Mudd JO Hiatt SO Lee CS. Identifying a relationship between physical frailty and heart failure symptoms. J Cardiovasc Nurs. (2018) 33(1):E1–7. 10.1097/JCN.0000000000000408 28353543 PMC5617768

[27] Denfeld QE Winters-Stone K Mudd JO Gelow JM Kurdi S Lee CS. The prevalence of frailty in heart failure: a systematic review and meta-analysis. Int J Cardiol. (2017) 236:283–9. 10.1016/j.ijcard.2017.01.153 28215466 PMC5392144

[28] Yang X Lupón J Vidán MT Ferguson C Gastelurrutia P Newton PJ Impact of frailty on mortality and hospitalization in chronic heart failure: a systematic review and meta-analysis. J Am Heart Assoc. (2018) 7(23):e008251. 10.1161/JAHA.117.008251 30571603 PMC6405567

[29] Sanders NA Supiano MA Lewis EF Liu J Claggett B Pfeffer MA The frailty syndrome and outcomes in the TOPCAT trial. Eur J Heart Fail. (2018) 20(11):1570–7. 10.1002/ejhf.1308 30225878

[30] Nelson JE Cox CE Hope AA Carson SS. Chronic critical illness. Am J Respir Crit Care Med. (2010) 182(4):446–54. 10.1164/rccm.201002-0210CI 20448093 PMC2937238

[31] Zannad F Ferreira JP Pocock SJ Anker SD Butler J Filippatos G SGLT2 inhibitors in patients with heart failure with reduced ejection fraction: a meta-analysis of the EMPEROR-reduced and DAPA-HF trials. Lancet. (2020) 396(10254):819–29. 10.1016/S0140-6736(20)31824-9 32877652

[32] Mone P Varzideh F Jankauskas SS Pansini A Lombardi A Frullone S SGLT2 inhibition via empagliflozin improves endothelial function and reduces mitochondrial oxidative stress: insights from frail hypertensive and diabetic patients. Hypertension. (2022) 79(8):1633–43. 10.1161/HYPERTENSIONAHA.122.19586 35703100 PMC9642044

[33] Abdelhafiz AH Sinclair AJ. Cardio-renal protection in older people with diabetes with frailty and medical comorbidities—a focus on the new hypoglycaemic therapy. J Diabetes Complicat. (2020) 34(9):107639. 10.1016/j.jdiacomp.2020.107639 32595017

[34] Sasaki T. Sarcopenia, frailty circle and treatment with sodium-glucose cotransporter 2 inhibitors. J Diabetes Investig. (2019) 10(2):193–5. 10.1111/jdi.12966 30369086 PMC6400153

[35] Sinclair AJ Pennells D Abdelhafiz AH. Hypoglycaemic therapy in frail older people with type 2 diabetes mellitus-a choice determined by metabolic phenotype. Aging Clin Exp Res. (2022) 34(9):1949–67. 10.1007/s40520-022-02142-8 35723859 PMC9208348

[36] Benjamin EJ Virani SS Callaway CW Chamberlain AM Chang AR Cheng S Heart disease and stroke statistics-2018 update: a report from the American heart association. Circulation. (2018) 137(12):e67–492. 10.1161/CIR.0000000000000558 29386200

[37] Loecker C Schmaderer M Zimmerman L. Frailty in young and middle-aged adults: an integrative review. J Frailty Aging. (2021) 10(4):327–33. 10.14283/jfa.2021.14 34549246

